# Effect of Early Nutritional Support on Clinical Outcomes of Critically Ill Patients with Sepsis and Septic Shock: A Single-Center Retrospective Study

**DOI:** 10.3390/nu14112318

**Published:** 2022-05-31

**Authors:** Jun-Kwon Cha, Hyung-Sook Kim, Eun-Ji Kim, Eun-Sook Lee, Jae-Ho Lee, In-Ae Song

**Affiliations:** 1 Department of Emergency Medicine, Seoul National University Bundang Hospital, Seongnam-si 13620, Korea; mira997@naver.com; 2 Inter-Department of Critical Care Medicine, Seoul National University Bundang Hospital, Seongnam-si 13620, Korea; kehese2956@gmail.com (H.-S.K.); rladmswl4626@naver.com (E.-J.K.); 3 Department of Pharmacy, Seoul National University Bundang Hospital, Seongnam-si 13620, Korea; escduck@gmail.com; 4 Division of Pulmonary and Critical Care Medicine, Department of Internal Medicine, Seoul National University Bundang Hospital, Seongnam-si 13620, Korea; jhlee7@snubh.org; 5 Division of Pulmonary and Critical Care Medicine, Seoul National University College of Medicine, Seoul 03080, Korea; 6 Department of Anesthesiology and Pain Medicine, Seoul National University Bundang Hospital, Seongnam-si 13620, Korea; 7 Department of Anesthesiology and Pain Medicine, Seoul National University College of Medicine, Seoul 03080, Korea

**Keywords:** enteral nutrition, parenteral nutrition, sepsis, septic shock

## Abstract

The initial nutritional delivery policy for patients with sepsis admitted to the intensive care unit (ICU) has not been fully elucidated. We aimed to determine whether an initial adequate nutrition supply and route of nutrition delivery during the first week of sepsis onset improve clinical outcomes of critically ill patients with sepsis. We reviewed adult patients with sepsis and septic shock in the ICU in a single tertiary teaching hospital between 31 November 2013 and 20 May 2017. Poisson log-linear and Cox regressions were performed to assess the relationships between clinical outcomes and sex, modified nutrition risk in the critically ill score, sequential organ failure assessment score, route of nutrition delivery, acute physiology and chronic health evaluation score, and daily energy and protein delivery during the first week of sepsis onset. In total, 834 patients were included. Patients who had a higher protein intake during the first week of sepsis onset had a lower in-hospital mortality (adjusted hazard ratio (HR), 0.55; 95% confidence interval (CI), 0.39–0.78; *p* = 0.001). A higher energy intake was associated with a lower 30-day mortality (adjusted HR, 0.94; 95% CI, 0.90–0.98; *p* = 0.003). The route of nutrition delivery was not associated with 1-year mortality in the group which was underfed; however, in patients who met > 70% of their nutritional requirement, enteral feeding (EN) with supplemental parenteral nutrition (PN) was superior to only EN (*p* = 0.016) or PN (*p* = 0.042). In patients with sepsis and septic shock, a high daily average protein intake may lower in-hospital mortality, and a high energy intake may lower the 30-day mortality, especially in those with a high modified nutrition risk in the critically ill scores. In patients who receive adequate energy, EN with supplemental PN may be better than only EN or PN, but not in underfed patients.

## 1. Introduction

Severe sepsis and septic shock remain frequent syndromes associated with high in-hospital mortality. A previous study reported that the mortality rate of patients with severe sepsis and septic shock was 40.4% [1]. Sepsis is a detrimental immune response to an infection that often induces an overwhelming reaction to abolish the normal reconstitution of immune cell homeostasis [2]. In sepsis, inflammatory mediators serve as potent inducers of catabolism; for example, cytokines play a critical role in breaking down proteins in muscles, promoting bone resorption, and driving lipolysis in adipocytes [3,4]. Further, inflammation-related endogenous skeletal muscle protein catabolism can quickly progress to severe muscle atrophy, especially early on in the process; it is also associated with immunosuppression, poor wound healing, intensive care unit (ICU)-acquired muscle weakness, and other adverse outcomes [1,3,4,5].

The early stages of catabolism in critically ill patients may explain why an early energy provision of up to 70%–80% of the measured energy expenditure is associated with improved outcomes [6,7]. Although the correct amount of protein needing to be administered to critically ill patients is unknown, doses higher than 1.2 g/kg are associated with reduced mortality in nonseptic nonenergy overfed patients in the ICU [6]. Furthermore, a recent large observational study suggested that achieving ≥80% of the prescribed protein intake in patients admitted to the ICU is associated with decreased mortality [8].

It has been claimed that nutrition therapy in patients with sepsis differs from standard nutritional approaches in critically ill patients. Although the causes for this remain unclear, it may be due to the infection-induced abnormal host responses that cause progressive physiologic alterations, which, consequently, limit metabolic capacity by impairing mitochondrial function [9].

Accordingly, the Surviving Sepsis Campaign guidelines do not recommend the early administration of parenteral nutrition (PN) with or without enteral nutrition (EN) in critically ill patients with sepsis and septic shock who can undergo enteral feeding over the first 7 days of sepsis onset [10,11,12]. In addition, the most recent SSC guidelines for 2021 proposed starting EN within 72 h in adult patients with sepsis or septic shock if possible as a weak grade [13]. However, few studies have investigated the use of exclusive or supplemental PN early in the acute phase of sepsis [14,15]. Moreover, Weijs et al. reported that an increased protein intake (1.2 g/kg/day) did not improve outcomes in patients with sepsis when compared with outcomes in patients without sepsis [6,16,17]; however, corresponding adverse effects were not observed. Moreover, a few well-established studies have investigated low-energy, low-protein nutrition for critically ill patients with sepsis.

One of the reasons for the aforementioned study results may be that it included both high-risk patients who required nutrition intervention and low-risk patients who did not. Therefore, in this study, the modified nutrition risk in critically ill (mNUTRIC) score was used to quantify the risk of patients developing worse clinical outcomes that may improve through intensive and adequate nutritional support; a high mNUTRIC score indicates that the patients achieved better clinical outcomes as a result of intensive nutritional support, while a low mNUTRIC score represents patients who received a minimal benefit from aggressive nutritional support [18].

In this study, we aimed to investigate the effects of nutrition, energy, protein supply, and route of nutrition delivery during the first 7 days of sepsis onset by assessing clinical prognosis markers, such as mortality, ventilator-free days, ICU length of stay (LOS), and hospital LOS. Additionally, the effects of nutritional support on clinical prognosis were examined separately in the high and low mNUTRIC score groups.

## 2. Methods

### 2.1. Ethics Approval and Consent to Participate

This retrospective study was approved by the Institutional Review Board of the Seoul National University Bundang Hospital (approval number: B-1907/550-101), and the requirement for written informed consent was waived.

### 2.2. Patient Population

We included patients aged ≥18 years who had an ICU LOS >3 days and experienced sepsis and septic shock in the ICU between 31st November 2013 and 20th May 2017 at the Seoul National University Bundang Hospital—a tertiary hospital. Patients who died or were discharged from our hospital within 3 days following sepsis onset, were pregnant, or had insufficient medical data to determine whether they had sepsis were excluded. Sepsis was diagnosed based on Sepsis-3 definition, which is ‘life-threatening organ dysfunction caused by a dysregulated host response to infection’. Organ dysfunction is defined as an acute change in the total sequential organ failure assessment (SOFA) score ≥2 points consequent to the infection [19]. Septic shock is defined as a clinical construct of sepsis with persisting hypotension requiring vasopressors to maintain mean blood pressure ≥65 mm Hg and having a serum lactate level >2 mmol/L (18 mg/dL) despite adequate volume resuscitation [19].

**Figure 1:**
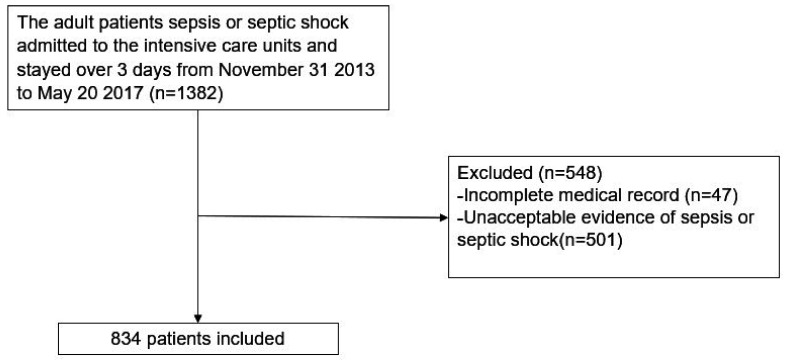
Patient selection flow chart.

Two intensivists reviewed the electronic medical records (EMR) of 1382 patients with suspected sepsis and septic shock from the documentation of health care providers or laboratory and physiological data, separately, and excluded 548 due to incomplete records or the absence of sepsis and septic shock. Finally, a total of 834 patients with sepsis and septic shock was enrolled in this study (Figure 1) [20].

### 2.3. Nutritional Support

The attending physician of the patients in the ICU determined the amount and type of nutrition and the route of nutrition delivery during the daily ICU rounds, with a nutrition support team (NST) comprised of intensivists, nurses, pharmacists, and nutritionists. NST provided detailed advice on nutritional support daily from the time patients were admitted to the ICU to when they were discharged. The attending physician or intensivists could consult with NST for further specific advice on nutrition support.

Nutrition targets were calculated by NST dietitians, pharmacists, and physicians using the Harrison–Benedict equation, stress factor adjustment, and daily protein requirements based on the American Society for Clinical Nutrition and Metabolism (ASPEN) and European Society for Clinical Nutrition and Metabolism (ESPEN) guidelines. The conditions of each patient, including sedation status, liver and renal function, and malnutrition status, were considered. After ICU admission, daily energy, protein delivery, and body weight were recorded. The ideal body weight (IBW) was calculated, and if the IBW was lower than the actual body weight (ABW), then the ABW was used. However, the adjusted body weight was calculated if the opposite was true.

After achieving hemodynamic stability, EN was preferred over PN. If enteral feeding was not feasible for any reason (such as gastric residual volume >500 mL, recurrent abdominal discomfort and distension, no bowel sounds, high vasopressor needs, aspiration events, gastrointestinal bleeding, etc.), PN was considered on days 3–5 of fasting or feeding intolerance. PN was reduced or delayed in patients with suspected liver failure, acute kidney injury (AKI), or biliary congestion. Protein intake was reduced if blood urea nitrogen or creatinine levels without renal replacement therapy increased steeply. Prokinetics were used to promote bowel movement.

### 2.4. Data Collection

Data on age, sex, height, weight, body mass index (BMI), daily energy and protein delivery, route of nutrition delivery, comorbidities, microbiological and laboratory results, number of days in which a mechanical ventilator was used, vasopressor use, in-hospital mortality, and ICU and hospital LOSs were collected via the EMRs. We calculated 30-day mortality rates following sepsis based on data obtained from EMR and the Ministry of the Interior and Safety.

The nutritional data of all patients were collected via EMRs, and the daily amounts of nutrition delivered via parenteral and enteral routes were recorded as daily total energy intake. Protein intake was recorded, and the daily achievement of an energy target (%) and daily average protein intake was calculated during the first week of sepsis onset. Moreover, the mNUTRIC scores, SOFA scores, and acute physiology and chronic health evaluation II (APACHE II) scores were calculated based on EMR data. The mNUTRIC scores were assessed to identify patients in the ICU with low mortality risk and a few days during which a mechanical ventilator was used due to aggressive nutritional therapy. This score included age, BMI, number of comorbidities, and illness severity (i.e., acute physiology and chronic health evaluation on APACHE II and SOFA scores) upon ICU admission.

### 2.5. Endpoint

The primary endpoint was in-hospital mortality following sepsis onset. Secondary endpoints were 30-day and 1-year mortality, number of ventilator-free days within 28 days of sepsis onset, and ICU and hospital LOSs following sepsis onset.

### 2.6. Statistical Analysis

SPSS version 24 (IBM Corp., Armonk, NY, USA) was used for all statistical analyses. Categorical variables were expressed as medians (interquartile range) and numbers (%), and continuous variables were expressed as means (standard deviation). The Kolmogorov–Smirnov test was used to determine the normality of the continuous variables.

Variance inflation factors (VIF) of independent variable were checked and only variables with VIF of less than 5 were included for regression analyses to avoid a multicollinearity [21]. All the independent variables included in this study demonstrated VIF of less than 4—low probability of interaction of variables [21].

Poisson log-linear regression analysis was used to determine the predictive factors associated with countable clinical outcomes, such as the number of ventilator-free days within 28 days of sepsis onset and ICU and hospital LOSs. Independent variables/confounders for adjustment included sex, mNUTRIC scores, SOFA scores, APACHE II scores, comorbidities, daily average achievement of an energy target (%), and average protein intake during the first week of sepsis onset [22]. Comorbidities are listed in Table 1 [22]. To eliminate bias, patients who died before ventilator cessation or who were discharged from the ICU or hospital were not included in this analysis.

A Cox regression analysis was used to determine whether mortality was associated with sex, mNUTRIC scores, SOFA scores, APACHE II scores, route of nutrition delivery, comorbidities, daily average achievement of an energy target, and average protein intake during the first week of sepsis onset. Univariable analyses were performed first, and factors with *p <* 0.05 were selected for multivariable analyses. In each multivariable analysis, we used a backwards stepwise regression approach to eliminate the predictors that did not independently contribute to 30-day and in-hospital mortalities. Odds ratios (ORs) and hazard ratios (HRs) with their 95% confidence intervals (CIs) were calculated. A *p*-value of <0.05 was considered statistically significant.

## 3. Results

A total of 834 patients in the ICU with sepsis and septic shock was included in this study. Among them, 467 patients had septic shock. The patients with septic shock were older and had higher SOFA scores, APACHE II scores, and mNUTRIC scores compared to patients with sepsis without septic shock. Further, they received prolonged ventilator care, high doses of vasopressors, and frequent, continuous renal replacement therapy (CRRT) compared to patients with sepsis without septic shock. Septic shock was associated with a prolonged hospital stay, prolonged ICU stay, and higher mortality among all patients with sepsis, as described in Table 1.

The demographic, physiologic, and nutritional data as well as clinical outcomes of all patients, are displayed in Table 1. The mean age was 68.9 years, and 65.5% of patients were male. The mean SOFA and APACHE II scores were 10.8 ± 3.7 and 32.0 ± 8.4, respectively. The mean number of days in which a mechanical ventilator was used was 8.4 ± 23.3 days, and the mean ICU and hospital LOSs were 13.2 ± 13.7 and 35.6 ± 42.3 days, respectively. In total, 200 patients with sepsis received CRRT. Among them, 176 patients who had not previously received RRT had AKI and underwent CRRT in the ICU [23].

The most common sources of infection were respiratory (57.6%), followed by the gastrointestinal entrance (15.1%). Furthermore, Gram-negative pathogens were the most common pathogens (32.3%). The in-hospital and 30-day mortality rates of all patients in this study were 33.7% (n = 281) and 28.8% (n = 240), respectively. The median mNUTRIC score was 6–8, and 89.9% of patients were in the high-score group (5–9 points). The median number of days on vasopressors, such as norepinephrine, was 2–8 days.

**Figure 2:**
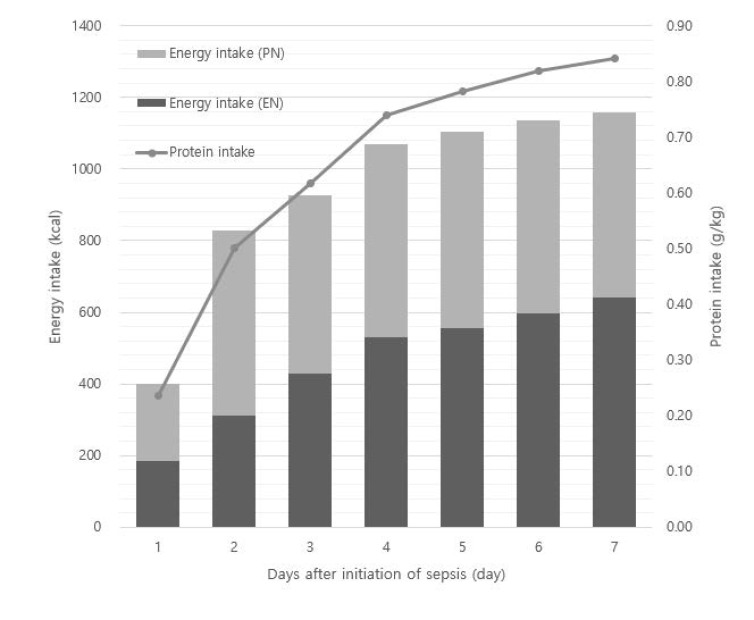
Daily energy (kcal) and protein (g/kg) received by patients following sepsis and septic shock onset. Column height represents the mean daily energy received (kcal). PN is the light grey and EN is the dark grey parts of the columns. Gray dots and lines represent protein intake (g/kg). EN, enteral nutrition; PN, parenteral nutrition.

Regarding the nutrition delivered during the first week of sepsis onset, average daily energy achievements of the target energy were 80.0 ± 33.7%, and the average daily protein intake was 0.64 ± 0.43 g/kg/day. Figure 2 shows the pattern of daily energy delivery (kcal) via enteral and parenteral feeding and daily protein intake (g/kg/day) during the first week of sepsis onset. There were no significant adverse events such as nutrition-induced sepsis, ischemic gut complications, and anastomotic leak; however, there were five cases of liver function test abnormality and one case of mild AKI.

### 3.1. Clinical Outcomes

#### 3.1.1. In-Hospital Mortality and Nutrition Supply

The association between in-hospital mortality and nutrition supply are outlined in Table 2. When analyzed using the univariable Cox regression analysis, the average daily protein and energy intake were significantly associated with reduced in-hospital mortality (HR, 0.43; 95% CI, 0.30–0.60; *p <* 0.001; and HR, 0.93; 95% CI, 0.90–0.96; *p <* 0.001, respectively). Increases in mNUTRIC (HR, 1.25; 95% CI, 1.16–1.36; *p <* 0.001), SOFA (HR, 1.14; 95% CI, 1.10–1.18; *p <* 0.001), and APACHE II scores (HR, 1.05; 95% CI, 1.04–1.07; *p <* 0.001) were associated with increases in in-hospital mortality. Sex and the number of comorbidities were not significantly associated with in-hospital mortality.

A multivariable Cox regression analysis was performed using the average daily protein and energy intake and mNUTRIC, SOFA, and APACHE II scores. The increased average daily protein intake during the first week of sepsis onset, mNUTRIC scores, and SOFA scores were associated with reduced in-hospital mortality (HR, 0.55; 95% CI, 0.39–0.78; *p* = 0.001; HR, 1.11; 95% CI, 1.01–1.23; *p* = 0.039; and HR, 1.07; 95% CI, 1.02–1.12; *p* = 0.006, respectively).

The results of the univariable Cox regression analysis revealed that no factor was associated with in-hospital mortality in the low mNUTRIC score group. Contrastingly, the average daily protein and energy intake were significantly associated with reduced in-hospital mortality in the high mNUTRIC score group (HR, 0.46; 95% CI, 0.32–0.65; *p <* 0.001; and HR, 0.93; 95% CI, 0.89–0.96; *p <* 0.001, respectively). Additionally, increases in the SOFA and APACHE II scores were associated with increases in in-hospital mortality (HR, 1.12; 95% CI, 1.08–1.16; *p <* 0.001; and HR, 1.05; 95% CI, 1.03–1.16; *p <* 0.001, respectively).

A multivariable Cox regression analysis was performed using the average daily protein and energy intake as well as SOFA and APACHE II scores. Increases in daily protein intake and SOFA and APACHE II scores during the first week of sepsis onset were associated with reduced in-hospital mortality in the high mNUTRIC score group (HR, 0.59; 95% CI, 0.42–0.84; *p* = 0.004; HR, 1.07; 95% CI, 1.02–1.12; *p* = 0.009; and HR, 1.03; 95% CI, 1.01–1.05; *p* = 0.015, respectively).

#### 3.1.2. Nutrition Supply and 30-Day Mortality

The association between 30-day mortality and nutrition supply is outlined in Table 3. The univariate Cox regression analysis showed that the average daily protein and energy intake, mNUTRIC scores, SOFA scores, APACHE II scores, and the number of comorbidities were significantly associated with a lower 30-day mortality. Further, increases in daily energy intake were associated with a lower 30-day mortality in the multivariable regression analysis after adjusting for mNUTRIC, SOFA, and APACHE II scores, as well as the number of comorbidities.

In the low mNUTRIC score group, there were no associations between the protein or energy intake and 30-day mortality. However, in the high mNUTRIC score group, the univariate regression analysis showed that both the daily energy and protein intake were associated with a lower 30-day mortality. Moreover, an increased average daily energy intake was associated with a reduced 30-day mortality in the high mNUTRIC score group, as revealed by the multivariate regression analysis (HR, 0.96; 95% CI, 0.92–1.00; *p* = 0.026).

Additionally, we compared the 30-day mortality of the septic shock and sepsis without septic shock groups (Table 4).

#### 3.1.3. Ventilator-Free Days within 28 Days of Sepsis Onset and Nutritional Support

The univariate Poisson log-linear regression analysis showed that the average daily protein and energy intakes were not significantly related to the number of ventilator-free days within 28 days of sepsis onset. The univariate Poisson log-linear analysis revealed that in the low mNUTRIC score group, the average daily protein and energy intakes were not significantly associated with the number of ventilator-free days within 28 days of sepsis onset. Contrastingly, patients in the high mNUTRIC score group had significantly longer ventilator-free days within 28 days of sepsis onset when a higher daily average energy intake was administered as assessed using the Poisson log-linear regression analysis. However, a multivariable Poisson log-linear regression analysis using the APACHE II and SOFA scores did not demonstrate the aforementioned association.

#### 3.1.4. Length of Stay in the ICU and Hospital and Nutritional Support

When analyzed using the univariate Poisson log-linear regression analysis, the average daily protein and energy intakes were significantly associated with reduced LOSs in the ICU (HR, 0.35; 95% CI, 0.22–0.54; *p <* 0.001; and HR, 0.90; 95% CI, 0.90–0.94; *p <* 0.001, respectively) and hospital (HR, 0.48; 95% CI, 0.34–0.67; *p <* 0.001; and HR, 0.93; 95% CI, 0.90–0.97; *p <* 0.001, respectively). However, a multivariate Poisson log-linear regression analysis showed that the amount of energy and protein intake was associated with a relatively low ICU LOS.

#### 3.1.5. Route of Nutrition Delivery and 1-Year Mortality

**Figure 3:**
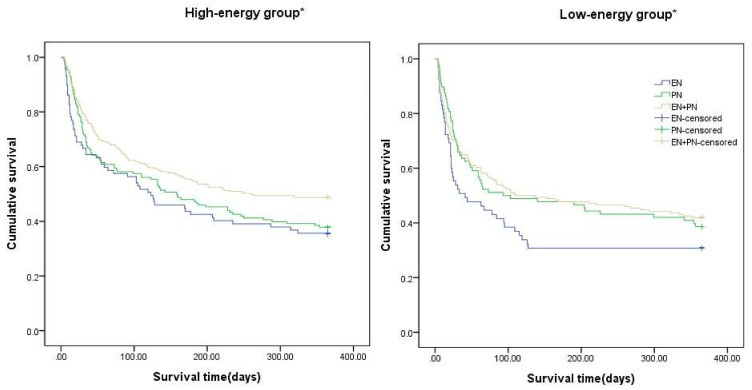
Kaplan–Meier curves for 1-year mortality according to the route of nutrition delivery in critically ill patients with sepsis in the low- and high-energy intake groups. * High-energy group, EN with supplemental PN was superior to only EN (*p* = 0.016) or PN (*p* = 0.042) in patients who met >70% of their nutrition requirement; low-energy group, route of nutrition supply was not associated with 1-year mortality in the group who met < 70% of their nutrition requirement. EN is the blue line, PN is the green line, and EN with supplemental PN is the purple line. EN, enteral nutrition; PN, parenteral nutrition.

The route of nutrition delivery during the first week of sepsis onset was associated with mortality (mortality in ICU (*p* = 0.002), in-hospital (*p* = 0.02), 30 day- (*p* = 0.002), and 1-year mortalities (*p* = 0,05)) in the result of a Kaplan–Meier estimates analysis. The EN-alone group looked to be associated with a higher ICU (*p* = 0.001), in-hospital (*p* = 0.006), 30-day (*p* = 0.021), and 1-year mortalities (*p* = 0.002) than the EN with supplemental PN group. Additionally, the EN-alone group showed higher ICU (*p* = 0.003) and 30-day mortalities (*p* = 0.001) than the PN-alone group. However, the route of nutrition delivery was not associated with mortality except for ICU mortality in the group that delivered <70% of their daily energy intake requirement. In the adequate nutrition group which met >70% of its patients’ daily energy intake requirement, the route of nutrition delivery was associated with ICU (*p* = 0.033), 30-day (*p* = 0.003) and 1-year mortalities (*p* = 0.005). Patients who were supplied with an adequate amount of energy via EN alone demonstrated less survival gain in the ICU (*p*= 0.013), during 30 days (*p* = 0.008), and 1 year following sepsis initiation (*p* = 0.016) than those who were treated via the EN with supplemental PN group, respectively. Especially, EN with supplemental PN seemed to increase the survival rate for 1 year in comparison to EN (*p* = 0.016) or PN alone (*p* = 0.042) in the adequate energy intake group (Figure 3).

What was special was that ICU mortality was associated with the route of nutrition administration in both groups which were underfed (*p* = 0.018) and those fed >70% of their daily energy intake requirement (*p* = 0.033). The EN-alone group showed a higher ICU mortality rate than EN with supplemental PN in the low caloric group (*p* = 0.001), and PN in the high-caloric group (*p* = 0.013), respectively.

The Cox regression analysis before adjustment also revealed that the EN-only group was related with a higher mortality (mortality in ICU (*p* = 0.001), in-hospital (*p* = 0.006), 30 day- (*p* = 0.001), and 1-year mortalities (*p* = 0.005)). An adjustment was performed for each mortality analysis with sex, APACHE II, SOFA, and mNUTRIC scores, the number of comorbidities, and the daily amount of energy and protein intake, respectively. After adjustment, the EN-alone group was associated with higher mortality in the ICU, hospital, 30-day, and 1-year mortalities. Table 5 revealed that the EN with supplemental PN group was superior to the EN-only group in improving the 1-year mortality during the first week of sepsis (*p* = 0.026).

## 4. Discussion

Although the crucial implications of nutritional supply in patients have been reported in many studies, information on the relationships between nutritional supply, the route of nutrition delivery, and prognosis in critically ill patients with sepsis and septic shock remains insufficient. The significance of this study is that it comprises 834 critically ill patients with sepsis and septic shock. In this study, protein intake increments of 0.1 g/kg/day during the first week of sepsis onset were associated with a 6% reduction in in-hospital mortality, especially in the high mNUTRIC score group. Additionally, increases in daily energy intake were associated with lower 30-day mortality, especially in the high mNUTRIC score group. Furthermore, EN with supplemental PN was superior to only EN in improving 1-year mortality during the first week of sepsis onset.

These findings might be new or different from previous studies [17,24]. The main reason could be a differences in the characteristics of patients enrolled in these studies. For instance, the patients in this study all had sepsis or septic shock; advanced age (median value: 72 years); high APACHE II, SOFA, and mNUTRIC scores (median values: 32, 11, and 7, respectively); and high in-hospital (33.7%) and 30-day mortality (28.8%). Contrastingly, other studies investigating nutrition in critically ill patients included fewer patients with sepsis (7–22%) and advanced age (<70 years) [24,25,26]. Furthermore, the participants of these studies had lower APACHE II, SOFA, and mNUTRIC scores (21–26, 8–9, and 4–5, respectively), and had an in-hospital mortality of 13%–48% [24,25,26].

While experts agree that the route of nutrition delivery matters, there is no consensus regarding which route is better for patients with sepsis in the ICU. Although the ASPEN guidelines suggest that critically ill patients should receive EN therapy within 24–48 h of confirming the diagnosis of severe sepsis or septic shock, avoiding the use of exclusive PN or supplemental PN in conjunction with EN early in the acute phase of severe sepsis or septic shock, there is no definite evidence to support the benefit of EN over PN according to mortality [27]. In this study, we divided the patients into three groups: (1) EN, (2) PN, and (3) EN with supplemental PN. We compared these three groups according to 1-year mortality, and the Cox-regression analysis revealed that EN with supplemental PN was superior to only EN in improving 1-year mortality during the first week of sepsis, even after adjusting the energy intake amount and patient severity scores. Further, Kaplan–Meier curves for 1-year mortality according to the route of nutrition delivery in the low- and high-energy intake groups showed that EN with supplemental PN was superior to only EN or PN in critically ill patients with sepsis who met >70% of their nutrition requirements (Figure 3). Contrastingly, the route of nutrition delivery was not associated with 1-year mortality in the group that met <70% of the nutrition requirement. These results suggest that EN alone could be harmful in septic patients with an adequate energy intake; however, the nutrition delivery route may not matter in patients who receive inadequate energy. The NUTRIREA-2 study, which enrolled patients with shock and high SOFA scores (11) and in-hospital mortality (36%), similar to this study, revealed that early EN did not show a reduced mortality or secondary infection compared to PN alone. However, this result cannot be directly applied to patients with sepsis exclusively; although the patients included in this study had similar patient severity to those included in our study, only 61%–64% of their patients had sepsis-related shock [27]. Moreover, in a multicenter randomized control trial including 33 English ICUs, Harvey et al. found no significant difference in 30-day mortality associated with the route of early nutritional support delivery in adult patients whose mean APACHE II score was 19, SOFA score was 9.5, and hospital mortality was approximately 36%–38%. However, this study did not include patients with sepsis [15].

Increases in daily energy intake were associated with a lower 30-day and in-hospital mortality before and after adjustment for patient severity scores and the amount of daily protein intake. A prospective study by Hung et al., including 151 patients with sepsis, found that patients with sepsis with an insufficient nutrition intake had a poor prognosis, despite being immunocompetent [28]. However, the number of patients included was small (low-energy (n = 16) vs. high-energy group (n = 69)). Additionally, the SOFA scores of the low-energy group were higher than those of the high-energy group; however, the study did not adjust for SOFA scores.

Increases in the daily protein intake were also associated with a reduced 30-day and in-hospital mortality before adjustment for patient severity scores and the amount of daily energy intake. A higher daily protein intake seemed to be related to improved outcomes upon discharge from the hospital after the abovementioned adjustments. For instance, a retrospective cohort study by Bendavid et al. revealed that an early protein provision in critically ill patients might be associated with improved survival, even after adjusting for confounders. Further, a single-center cohort study by Weijs et al. also showed that patients in the ICU with improvements in the daily protein intake during hospitalization had decreased odds of mortality in the three months following hospital discharge. However, in both studies, patients with sepsis only comprised 17% and 21% of the study population, respectively [3,25].

An important characteristic of patients in this study was that they were not overfed; the average amount of energy delivered during the first week of sepsis was, in median values, 923.6 (663.7–1165.1) kcal and 80.0% of the basal energy expenditure. Additionally, the average protein supply during the first week following sepsis diagnosis was 0.62 g/kg/day, which is lower than the protein limit of 0.8 g/kg/day recommended by the ASPEN guidelines [15,25]. In the Augmented versus Routine approach to Giving Energy Trial (TARGET), participants randomly assigned to the 1.5 kcal/mL group received approximately 1900 kcal per day on average, whereas those assigned to the 1.0 kcal/mL group received approximately 1300 kcal per day on average. An increased EN caloric feeding due to an energy-dense nutrient intake did not affect the 90-day mortality, survival time, ICU and hospital discharge days, or the incidence of infective complications [29]. Thus, EN is generally limited in critically ill patients with sepsis due to excessive vasopressor use [21], as ischemia of the bowels can occur due to the shock state. Second, supplemental PN is often not recommended or allowed in cases of multiorgan failure. Second, supplemental PN is often not recommended or allowed in cases of multiorgan failure, including haptic or renal injury. Finally, we also determined the amount of energy with reference to the ASPEN/Society of Critical Care Medicine or ESPEN guidelines [12,27]. The ASPEN/Society of Critical Care Medicine guidelines recommend increasing the amount of energy during the first week after initially attempting trophic feeding for critically ill patients with sepsis [25]. Notably, the ESPEN guidelines recommend low-calorie nutrition at a level of 70% (25–30 kcal/kg/day) for critically ill patients with sepsis [11]. This should be clarified in future studies via randomized controlled trials.

Our study had several limitations. First, this was a relatively small single-center study. However, we achieved statistical significance and used strict selection criteria. Second, as data on patients were obtained over a 3-year period, which is a relatively long period, management and faculty may have changed; this is significant because nutritional support aggressiveness depends on the attending physician’s decision and discretion. Generally, an NST also conducts regular ICU rounds and provides continuous guidance on the nutrition plan. Therefore, the nutritional support provided in the ICU may have remained relatively consistent. The third limitation was the presence of unavoidable bias due to the retrospective nature of the study.

## 5. Conclusions

In critically ill patients with sepsis, the mortality decreased as the amount of protein or energy supply increased during the first week of sepsis onset. Additionally, for patients with sepsis in the ICU, EN with supplemental PN may be a better route of nutrition delivery than EN or PN alone.

## Figures and Tables

**Table 1:** Baseline characteristics and clinical outcomes of patients.

Characteristic	Total (n = 834)	Septic Shock (n = 467)	Sepsis without Septic Shock (n = 367)	*p*-Value
Sex, male	546 (66%)	303 (65%)	243 (73%)	0.714
Age (years)	72 (62–78)	72 (63–78)	73 (60–79)	0.795
BMI (kg/m^2^)	21 (18–24)	22 (18–24)	21 (18–24)	0.091
SOFA score	11 (9–13)	12 (10–14)	9 (6–12)	<0.001 *
APACHE II score	32 (26–38)	35 (29–40)	29 (23–24)	<0.001 *
mNUTRIC scores	7 (6–8)	7 (6–8)	7 (5–8)	<0.001 *
Low score (0–4 points)	84 (10.1%)	20 (4.3%)	64 (17.4%)	
High score (5–9 points)	750 (89.9%)	447 (95.7%)	303 (82.6%)	
Comorbidities	2.5 (1.7)	2.5 (1.7)	2.4 (1.7)	0.661
Myocardial disease	216 (25.9%)	127 (27%)	89 (24%)	
Peripheral vascular disease	438 (52.5%)	249 (53%)	189 (24%)	
Pulmonary disease	139 (16.7%)	63 (13%)	76 (51%)	
Neurologic disease	161 (19.3%)	75 (16%)	86 (23%)	
Endocrinal disease	233 (27.9%)	145 (31%)	88 (24%)	
Chronic renal disease	108 (12.9%)	63 (13%)	45 (12%)	
Previous RRT	41 (4.9%)	23 (5%)	18 (5%)	
Gastrointestinal disease	82 (9.8%)	55 (12%)	27 (7%)	
Cancer/immunocompromised state	267 (32.0%)	157 (34%)	110 (30%)	
Psychological disease	24 (2.9%)	17 (4%)	7 (2%)	
Musculoskeletal disease	86 (10.3%)	51 (11%)	35 (10%)	
Substance use disorder	27 (3.2%)	11 (2%)	16 (4%)	
Miscellaneous	26 (3.1%)	17 (4%)	9 (2%)	
Types of intensive care units (ICUs)				<0.001 *
Medical ICU	578 (69.3%)	327 (70%)	251 (68%)	
Surgical ICU	103 (12.4%)	71 (15%)	32 (9%)	
Emergency ICU	93 (11.2%)	58 (12%)	35 (10%)	
Neuro/neurosurgical ICU	60 (7.2%)	11 (2%)	49 (13%)	
Source of infection				<0.001 *
Respiratory infection	480 (57.6%)	227 (49%)	253 (69%)	
Genitourinary infection	41 (4.9%)	26 (6%)	14 (4%)	
Gastrointestinal infection	126 (15.1%)	97 (21%)	29 (8%)	
Other infections	115 (13.8%)	72 (15%)	44 (12%)	
Multiple infections	53 (6.4%)	27 (6%)	16 (4%)	
Unknown source of infection	19 (2.3%)	18 (4%)	1 (0%)	
Pathogen type				0.039 *
Gram-positive pathogens	176 (21.1%)	96 (21%)	80 (22%)	
Gram-negative pathogens	269 (32.3%)	167 (36%)	102 (28%)	
Other	12 (1.4%)	6 (1%)	60 (16%)	
Multimicrobial infections	125 (15.0%)	70 (15%)	55 (15%)	
Fungi	30 (3.6%)	18 (4%)	12 (3%)	
Viruses	27 (3.2%)	8 (2%)	19 (5%)	
Unidentified pathogens	195 (23.4%)	102 (22%)	93 (25%)	
Nutrition †				
Protein intake per body weight (g/kg/day) †	0.6 (0.4–0.9)	0.6 (0.3–0.8)	0.7 (0.5–1.0)	<0.001 *
Protein intake (g/day) †	34.9 (18.7)	31.3 (18.7)	39.3 (17.8)	<0.001 *
Energy intake (kcal/day) †	926.0 (377.2)	867.2 (36.2.5)	1000.9 (382.7)	<0.001 *
Target energy achievement (%) †	80.0 (33.7)	74.5 (32.0)	87.0 (34.6)	<0.001 *
Treatment in ICU				
Vasopressor use days	5 (2–8)	6 (4–11)	2 (0–6)	<0.001 *
Ventilator-free days during 28 days	23 (14–26)	24 (18–28)	26 (21–28)	0.005
New continuous renal replacement therapy	200 (24.0%)	166 (36%)	34 (9%)	<0.001 *
Clinical outcomes				
Length of ICU stay (days)	9 (6–15)	9 (6–16)	8 (6–14)	<0.001 *
Length of hospital stay (days)	25 (14–43)	24 (14–42)	26 (15–44)	0.137
ICU mortality	175 (21.0%)			
In-hospital mortality	281 (33.7%)	201 (43%)	80 (22%)	<0.001 *
30-day mortality	240 (28.8%)	163 (35%)	77 (21%)	<0.001 *
1-year mortality	488 (58.5%)	306 (66%)	182 (50%)	<0.001 *
Discharge course				<0.001 *
Other hospital	211 (25.3%)	104 (22%)	107 (29%)	
Healthcare center	58 (7.0%)	27 (6%)	31 (8%)	
Home	276 (33.1%)	130 (28%)	146 (40%)	
Death	280 (33.6%)	201 (43%)	79 (22%)	
Other courses	9 (1.1%)	5 (1%)	4 (1%)	

Numbers are presented as median (interquartile range) or numbers (percentages). * *p <* 0.05 was considered statistically significant; † average daily amount of nutrition (energy or protein) during the first week after sepsis onset. BMI, body mass index; SOFA, sequential organ failure assessment score; APACHE II, acute physiology and chronic health evaluation II; ICU, intensive care unit; BMI, body mass index; mNUTRIC, modified nutrition risk in the critically ill.

**Table 2:** Association between in-hospital mortality and nutrition supply in patients with sepsis one week after sepsis onset.

	Total (n = 834)				mNUTRIC Scores							
					Low (n = 84)				High (n = 750)			
	Unadjusted		Adjusted †		Unadjusted		Adjusted †		Unadjusted		Adjusted †	
	Hazard Ratio (95% CI)	*p*-Value	Hazard Ratio (95% CI)	*p*-Value	Hazard Ratio (95% CI)	*p*-Value	Hazard Ratio (95% CI)	*p*-Value	Hazard Ratio (95% CI)	*p*-Value	Hazard Ratio (95% CI)	*p*-Value
Sex, male	1.11 (0.86–1.43)	0.412			0.77 (0.18–3.28)	0.727			1.11 (0.86–1.44)	0.413		
SOFA score	1.14 (1.10–1.18)	<0.001 *	1.07 (1.02–1.12)	0.006 *	1.15 (1.00–1.34)	0.065			1.12 (1.08–1.16)	<0.001 *	1.07 (1.02–1.12)	0.009 *
APACHE II score	1.05 (1.04–1.07)	<0.001 *	1.02 (1.00–1.04)	0.083	1.00 (0.89–1.12)	0.971			1.05 (1.03–1.06)	<0.001 *	1.03 (1.01–1.05)	0.015 *
Comorbidities	1.06 (1.00–1.13)	0.070			1.01 (0.61–1.68)	0.975			1.03 (0.96–1.10)	0.457		
Protein intake (g/kg/day) †	0.43 (0.30–0.60)	<0.001 *	0.55 (0.39–0.78)	0.001 *	0.22 (0.03–1.50)	0.121			0.46 (0.32–0.65)	<0.001 *	0.59 (0.42–0.84)	0.004 *
Energy intake, target 10% †	0.93 (0.90–0.96)	<0.001 *			0.88 (0.71–1.09)	0.250			0.93 (0.89–0.96)	<0.001 *		
mNUTRIC score	1.25 (1.16–1.36)	<0.001 *	1.11 (1.01–1.23)	0.039 *								

† Average daily amount of nutrition during the first week following sepsis onset. CI, confidence interval; mNUTRIC, modified nutrition risk in the critically ill; SOFA, sequential organ failure assessment score; and APACHE II, acute physiology and chronic health evaluation II. * *p <* 0.05 was considered statistically significant; † A multivariable Cox regression analysis was performed using risk factors (*p <* 0.05) identified from the univariable Cox regression analysis.

**Table 3:** Association between 30-day mortality and nutrition supply one week after sepsis or septic shock onset.

	Total (n = 834)				mNUTRIC Score							
					Low (n = 84)				High (n = 750)			
	Unadjusted		Adjusted †		Unadjusted		Adjusted †		Unadjusted		Adjusted †	
	Hazard Ratio (95% CI)	*p*-Value	Hazard Ratio (95% CI)	*p*-Value	Hazard Ratio (95% CI)	*p*-Value	Hazard Ratio (95% CI)	*p*-Value	Hazard Ratio (95% CI)	*p*-Value	Hazard Ratio (95% CI)	*p*-Value
Sex, male	1.17 (0.89–1.54)	0.249			1.30 (1.10–1.53)	0.002 *	1.28 (1.08–1.53)	0.006 *	1.13 (1.09–1.18)	<0.001 *	1.07 (1.02–1.13)	0.010 *
SOFA score	1.06 (1.05–1.08)	<0.001 *	1.07 (1.02–1.13)	0.009 *	1.06 (0.92–1.21)	0.390			1.05 (1.04–1.07)	<0.001 *	1.03 (1.01–1.06)	0.003 *
APACHE II score	1.36 (1.24–1.50)	<0.001 *	1.02 (1.00–1.05)	0.049 *	0.89 (0.43–1.83)	0.749			1.05 (0.98–1.13)	0.184		
Comorbidities	1.09 (1.02–1.17)	0.016 *			0.10 (0.01–2.03)	0.132			0.51 (0.35–0.73)	<0.001 *		
Protein intake (g/kg/day) **	0.47 (0.32–0.68)	<0.001 *			0.70 (0.49–0.99)	0.043 *	0.71 (0.48–1.05)	0.084	0.93 (0.89–0.97)	<0.001 *	0.96 (0.92–1.00)	0.026 *
Energy intake, target 10% **	0.92 (0.89–0.96)	<0.001 *	0.94 (0.90–0.98)	0.003 *	0.98 (0.18–5.36)	0.983			1.19 (0.90–1.57)	0.220		
mNUTRIC score	1.16 (1.12–1.20)	<0.001 *	1.21 (1.08–1.36)	0.001 *								

CI, confidence interval; mNUTRIC, modified nutrition risk in the critically ill; SOFA, sequential organ failure assessment score; APACHE II, acute physiology and chronic health evaluation II. * *p <* 0.05 was considered statistically significant. † Multivariable Cox regression was performed with risk factors (*p <* 0.05) identified from the univariable Cox regression analysis. ** Average daily amount of nutrition (protein and energy) during the first week following sepsis initiation.

**Table 4:** Subgroup analysis for association between 30-day mortality and nutrition supply of the septic shock and sepsis without septic shock groups.

	Septic Shock (n = 467)				Sepsis without Shock (n = 367)			
	Unadjusted		Adjusted †		Unadjusted		Adjusted †	
	Hazard Ratio (95% CI)	*p*-Value	Hazard Ratio (95% CI)	*p*-Value	Hazard Ratio (95% CI)	*p*-Value	Hazard Ratio (95% CI)	*p*-Value
Sex, male	0.93 (0.67–1.29)	0.657			0.68 (0.41–1.13)	0.135		
SOFA score	1.14 (1.08–1.20)	<0.001 *	1.06 (1.04–1.16)	0.001 *	1.16 (1.08–1.23)	<0.001 *		
APACHE II score	1.05 (1.03–1.07)	<0.001 *			1.07 (1.04–1.10)	<0.001 *	1.04 (1.00–1.08)	0.047 *
Comorbidities	1.07 (0.98–1.17)	0.169			1.11 (0.98–1.26)	0.088		
Protein intake (g/kg/day) **	0.57 (0.36–0.91)	0.018 *			0.51 (0.26–0.99)	0.045 *		
Energy intake, target 10% **	0.99 (0.98–0.99)	0.012 *	0.99 (0.98–0.99)	0.03 *	0.99 (0.99–1.00)	0.06		
mNUTRIC score	1.32 (1.16–1.51)	<0.001 *	1.25 (0.09–1.44)	0.002 *	1.37 (1.19–1.59)	<0.001 *	1.21 (1.00–1.46)	0.048 *

CI, confidence interval; mNUTRIC, modified nutrition risk in the critically ill; SOFA, sequential organ failure assessment score; APACHE II, acute physiology and chronic health evaluation II. * *p <* 0.05 was considered statistically significant. † Multivariable Cox regression was performed with risk factors (*p <* 0.05) identified from the univariable Cox regression analysis. ** Average daily amount of nutrition (protein and energy) during the first week following sepsis onset.

**Table 5:** Association between nutrition delivery route and 1-year mortality for patients with sepsis or septic shock.

	Total (n = 827)			
	Unadjusted		Adjusted †	
	Hazard Ratio (95% CI)	*p*-Value	Hazard Ratio (95% CI)	*p*-Value
Sex, male	1.29 (1.07–1.57)	0.009 *	0.75 (0.62–0.92)	0.004 *
SOFA score	1.10 (1.08–1.13)	<0.001 *		
APACHE II score	1.05 (1.04–1.06)	<0.001 *	1.03 (1.02–1.05)	<0.001 *
Comorbidities	1.12 (10.7–1.18)	<0.001 *	1.06 (1.01–1.13)	0.034 *
mNUTRIC score	1.25 (1.18–1.33)	<0.001 *	1.11 (1.03–1.21)	0.008 *
Energy, target 10%	0.97 (0.94–0.99)	0.013 *	0.97 (0.94–1.00)	0.032 *
Route of nutrition delivery		0.005 *		0.067
EN	1.46 (1.16–1.84)	<0.001 **	1.31 (1.03–1.65)	0.026 **
PN	1.19 (0.97–1.47)	0.093	1.16 (0.94–1.42)	0.171
EN with supplemental PN	1		1	

CI, confidence interval; mNUTRIC, modified nutrition risk in the critically ill; SOFA, sequential organ failure assessment score; APACHE II, acute physiology and chronic health evaluation II, EN, enteral feeding; PN, parenteral feeding. * *p <* 0.05 was considered statistically significant. ** *p <* 0.05 compared to EN with supplemental PN. † Multivariable Cox regression was performed with risk factors (*p <* 0.05) identified from the univariable Cox regression analysis.

## Data Availability

Data available on request due to restrictions e.g., privacy or ethical. The data presented in this study are available on request from the corresponding author. The data are not publicly available due to privacy policy of our hospital.
